# Fibrin as a Multipurpose Physiological Platform for Bone Tissue Engineering and Targeted Delivery of Bioactive Compounds

**DOI:** 10.3390/pharmaceutics11110556

**Published:** 2019-10-28

**Authors:** Bruno Bujoli, Jean-Claude Scimeca, Elise Verron

**Affiliations:** 1 Chemical and Interdisciplinarity, Synthesis, Analysis, Modelisation, CEISAM UMR CNRS 6230, University of Nantes, 44300 Nantes, France; bruno.bujoli@univ-nantes.fr; 2 Université Côte d’Azur, CNRS, Inserm, iBV, 06107 Nice, France; jean-claude.scimeca@unice.fr

**Keywords:** fibrin, natural polymers, biomaterials, delivery systems, bone regeneration

## Abstract

Although bone graft is still considered as the gold standard method, bone tissue engineering offers promising alternatives designed to mimic the extracellular matrix (ECM) and to guide bone regeneration process. In this attempt, due to their similarity to the ECM and their low toxicity/immunogenicity properties, growing attention is paid to natural polymers. In particular, considering the early critical role of fracture hematoma for bone healing, fibrin, which constitutes blood clot, is a candidate of choice. Indeed, in addition to its physiological roles in bone healing cascade, fibrin biochemical characteristics make it suitable to be used as a multipurpose platform for bioactive agents’ delivery. Thus, taking advantage of these key assets, researchers and clinicians have the opportunity to develop composite systems that might further improve bone tissue reconstruction, and more generally prevent/treat skeletal disorders.

## 1. Introduction

Bone is a dynamic tissue resulting from a permanent remodeling process under the control of bone-forming cells (i.e., osteoblasts) and bone-resorbing cells (i.e., osteoclasts). Thanks to this property, the healing process can restore both biological and mechanical functions of bone in case of a non-critical size defect [1]. However, in addition to size-related issues (critical defects which cannot be filled with bone autograft), bone repair can be disturbed for reasons linked to age, individual behavior (i.e., smoking, inactivity) and diseases (i.e., bone infections, tumors, osteoporosis). This prompted the development of bone substitutes, available in large quantities and suitable for use in harsh bone repair conditions. Thanks to a better understanding of molecular and cellular mechanisms underlying bone regeneration, sophisticated biomaterials adapted for bone tissue engineering have been developed [2,3]. In line with the growing development of bio-inspired materials, natural polymers have attracted much attention, due to their similarity to the extracellular matrix (ECM), as well as low toxicity/immunogenicity properties. Among them, a special attention has been given to fibrin, and this review aims at providing the reader with a set of characteristics explaining the relevance of fibrin choice for bone substitutes designing.

## 2. Fibrin Clot Action within Bone Healing Process

**Figure 1:**
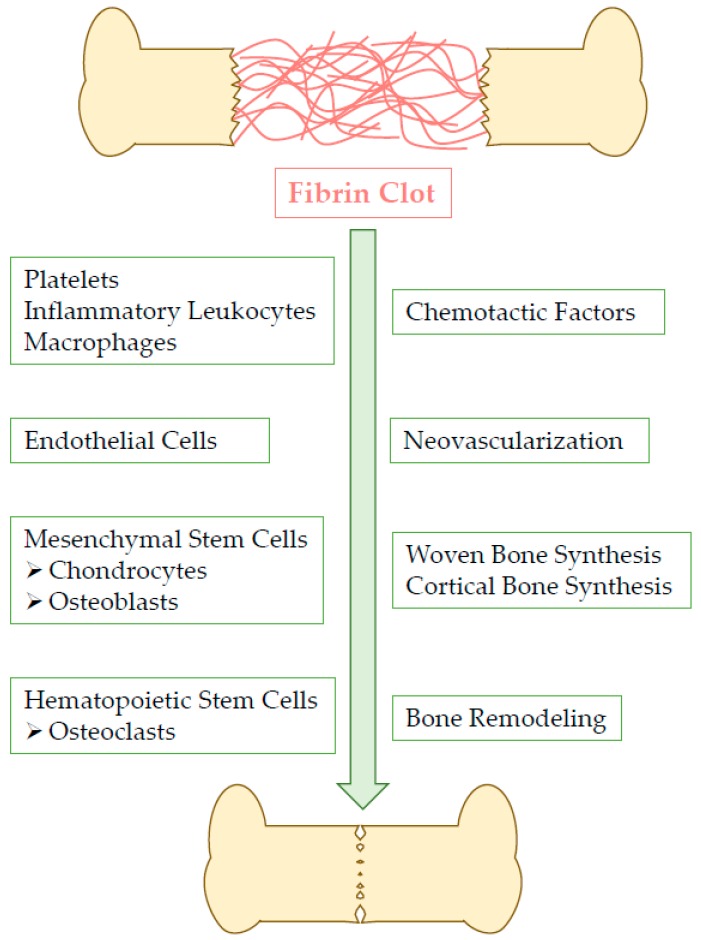
Fibrin clot-induced physiological bone healing cascade [4].

As depicted in Figure 1, fibrin constitutes the blood clot, the basic structure of fracture hematoma, and it provides a temporary matrix for wounded tissue until complete remodeling. Fibrin also promotes angiogenesis and osteogenic differentiation, hence favoring bone regeneration [4,5].

In addition to that, fibrin displays particular features which offer the prospect of using it as a versatile platform for the delivery of various agents favoring bone reconstruction in critical situations. Thus, fibrin can be considered as a promising candidate capable to play a key dual role: (i) enable physiological bone repair for critical defects; (ii) deliver bio-active compounds in particularly adverse conditions.

After reminding fibrin physical and biological properties, this review will describe strategies to tailor/tune and regulate the release profile of entrapped therapeutic compounds. Lastly, the broad range of fibrin-based strategies will be illustrated by various examples related to the local delivery of drugs, growth factors, genes and miRNAs.

## 3. Fibrin Physiological Properties

### 3.1. Composition, Structure, and Mechanical Properties

Fibrinogen is an elongated protein, 45 nm in length, which is made up of globular domains at each end connected by alpha-helical coiled-coils to a globular region in the middle. Circulating fibrinogen is cleaved by the action of the serine protease thrombin, resulting in the formation of an insoluble three-dimensional network capable to entrap molecules and cells [6]. At sites of wound healing and angiogenesis, transglutaminase factor XIII (F XIIIa) activation leads to the deposition and the stabilization of fibrin clots. Once stabilized, these fibrin clots serve as a temporary matrix that provides solid supports for invading fibroblasts and endothelial cells, before undergoing an enzymatic degradation concomitant to the regeneration of damaged tissues [7].

Fibrin network formation must be tightly controlled as it influences the clot properties. Indeed, various parameters influence the fibrin mesh thickness, porosity and permeability. In that respect, thrombin, fibrinogen, H^+^ and Ca^2+^ concentrations, temperature, as well as the presence of hyaluronic acid and plasma proteins, deeply affect the network microstructure [6,8,9]. For example, high thrombin concentrations favor the formation of clot with thin fibers and small pores, whereas lower concentrations tend to create clot with thick fibers and large pores [10]. The fibrinogen concentration also regulates clot properties. Under physiological conditions (fibrinogen at a concentration of 2.5 g/L), plasma will rapidly clot to form a mechanically stable structure, with large spaces between protofibrils, and much larger spaces between fibers. In this structure, fibrin represents only 0.25% of the volume while 99.75% of the clot is made of fluid occupying the space between protein polymers [11]. Considering the small amount of material involved, fibrin networks display remarkable properties.

Fibrin clot mechanical properties are extremely important for numerous clinical phenomena including hemostasis, fibrinolysis, thrombus deformation in the blood flow and embolization. Consequently, based on theoretical and experimental data, and in order to investigate the behavior of fibrin networks upon mechanical deformations (shear deformation, strain stiffening etc.), a number of models have been developed [12,13,14,15]. Among them, one is based on the viscoelastic properties of fibrin. It demonstrates that its mechanical response depends on the duration and the rate of loading [16,17]. As deeply described by Weisel et al. [11], stress seems directly proportional to strain at low strains whereas fibrin stiffness increases until 20-fold at larger strains. The authors suggest that fibrin network expresses irreversible deformation but surprisingly, it can completely recover its stiffness after removing the stress. Furthermore, fibrin has been shown to display a nonlinear mechanical response to external compression [14]. Kim et al. define the three following distinct regimes (i) a linear viscoelastic response to compression resulting in fibers become straight; (ii) a stress plateau where fibers buckle and collapse, and (iii) a network densification characterized by a stress-strain nonlinear response and dominated by bending of fibers after inter-fiber contact. Lastly, fibrin deformation occurs non-homogeneously along the matrix [18]. To sum up, all these properties influence clot responses to forces such as stretching, bending or buckling [17].

### 3.2. Biological Properties

In addition to these mechanical characteristics, fibrin displays at least two major biological properties. First of all, it participates to hemostasis in wound healing. In addition, the blood clot specifically interacts with numerous ECM proteins and growth factors such as fibronectin, vitronectin, fibroblast growth factor (FGF), vascular endothelial growth factor (VEGF), insulin-like growth factor-1 (IGF-1), and enzymes including plasminogen or tissue plasminogen activator [6]. By consequence, fibrin provides also many cell types with a provisional matrix which supports tissue remodeling and healing.

**Figure 2:**
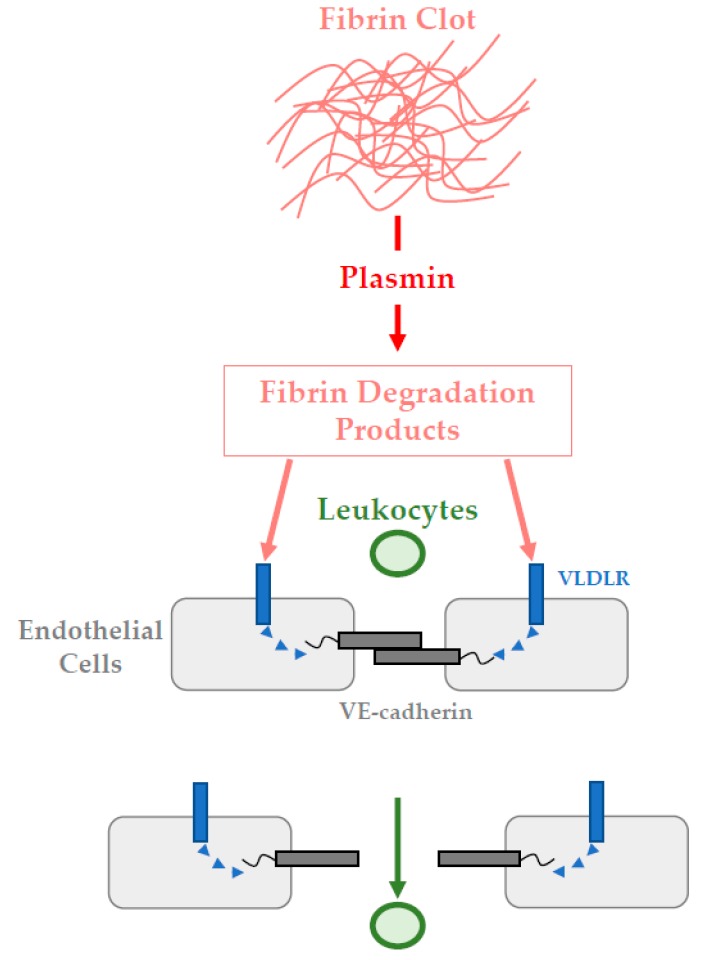
Fibrin clot degradation, and leukocytes transmigration triggering inflammatory response onset.

Within the specific context of bone repair, fibrin exerts a key role at the beginning of the process. As mentioned above, an initial inflammatory response is required and produces chemotactic factors which attract cells involved in tissue reconstruction. Figure 2 below illustrates how fibrin participates to this critical step. Indeed, fibrin degradation products directly act on endothelial cells forming vascular walls. Through a mechanism involving VLDL receptor and VE-cadherin junction proteins, leukocytes transmigration occurred from surrounding vessels towards injured sites where they contribute to the inflammatory response onset [19,20].

As illustrated above and under physiological conditions, the blood clot is not a permanent structure. Fibrinogenesis and blood coagulation are counteracted by the fibrinolytic system in order to preserve the hemostatic balance [21]. Regulating this balance is crucial to drive tissue formation and neovascularization while allowing a progressive degradation of fibrin clot. When fibrin degradation occurs too fast, which is observed for example at low concentration of fibrinogen (e.g., 9 mg/mL) and thrombin (50 U/mL), osteogenic cells are not able to differentiate and produce their own matrix to fill the defect site [22]. These negative effects can be reversed by increasing the content of fibrin constituents. However, it is worth noting that fibrinogen and thrombin must be kept at physiological concentrations, which provide a slow gelation time essential to protect entrapped cells from osmotic shock and mechanical stress, and to preserve their differentiation potential.

A large set of data further support the notion that fibrin clot characteristics and its interactions with cells are tightly linked. For example, the formation of dense hydrogels by increasing fibrinogen concentration results in an inhibition of cells migration and proliferation [23,24], while it increases their osteogenic differentiation, probably in response to the high concentration of growth factors trapped inside the material [25]. The fibrinogen concentration also influences cells morphology. At high fibrinogen concentration (50 mg/mL), cells are rounded whereas they appear elongated and spindle-like at low fibrinogen concentration (5 mg/mL) [26]. Moreover, sodium chloride supplementation during gel fabrication increases the ionic strength, and contributes to maintaining the viability and the osteogenic potential of entrapped cells [27].

Regarding in vitro cell culture, different surface treatments have been investigated. In this way, precoating cell culture plates with 10 mg/mL fibrinogen promotes mesenchymal stem cells (MSC) adhesion, proliferation and differentiation [28]. When compared to cells cultured directly on tissue culture plastic, MSC preculture during 7 days on a fibrin matrix before re-seeding them onto tissue culture plastic enhances their proliferation and their osteogenic properties [29]. Oh et al. cultured MC3T3-E1 pre-osteoblasts on fibrin matrices prepared with various concentrations of thrombin, and they reported that thrombin promotes fibrin-enhanced osteoblast differentiation in a dose-dependent manner [30]. Furthermore, as regards various cells including fibroblasts, modulating the plasma-derived fibrin concentration influences cellular characteristics including proliferation rate, migration, ECM production and soluble factors secretion [31].

## 4. Preparing Fibrin for Bone Repair

Fibrin-derived products can be industrially manufactured using large plasma pools and fractionation/precipitation steps, which include cryoprecipitation and ethanol fractionation [32,33,34]. These products contain concentrated fibrinogen (>80 g/L) and purified thrombin (500–1000 UI/mL) obtained from the activation of prothrombin [35]. This manufacturing process leads also to the purification of other plasma proteins, and growth factors concentration can be modified according to the method used. Calcium chloride is usually added to accelerate fibrin polymerization. The fibrinogen and thrombin components are mixed extemporaneously to form, within seconds, a fibrin clot that adheres to tissues, mimicking the last step of the coagulation cascade. If it is necessary to slow down fibrinolysis, formulations include antifibrinolytic agents, such as tranexamic acid, or low concentration of thrombin (<10 IU/mL). As largely described in the literature, different fibrin glues with diverse structures and mechanical/stiffness properties are commercially available [36]. Finally, regarding clinical needs/outcomes, various applicators including sprays, double-syringes and endoscopic devices are also on the market.

Fibrinogen-enriched products can also be prepared from single donations, mainly by using cryoprecipitation. Briefly, cryoprecipitate isolated by centrifugation is suspended at room temperature with residual cryo-poor plasma and used fresh or after freezing/thawing. It is considered that 200 mL of plasma provide approximately 15 mL of cryoprecipitate. The thrombin stability can be enhanced by adding ethanol (10–15%) [37].

**Figure 3:**
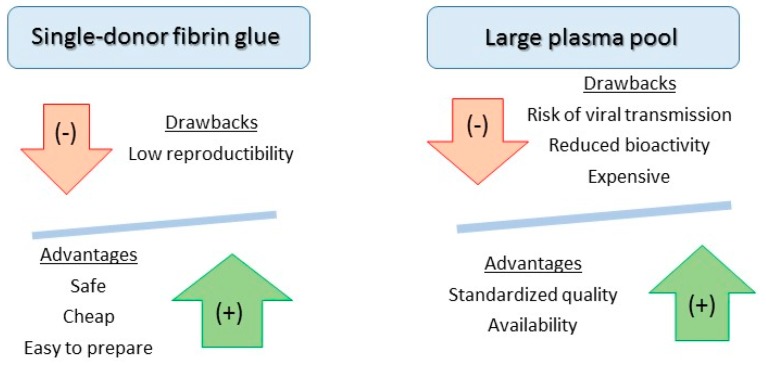
Main advantages and drawbacks according to the origin of fibrin glue.

The selection of appropriate fibrin glue is mainly based on criteria such as ease of preparation, safety, reproducibility and cost. Although single-donor fibrin is cheap and safe as compared to commercial fibrin, it lacks satisfactory reproducibility. However, it can be prepared easily from platelet-rich plasma, which constitutes a concentrated source of growth factors supporting bone regeneration. As a matter of fact, platelet-rich fibrin mixed with inorganic bovine bone particles (Bio-Oss^®^) significantly enhances bone augmentation in the maxillary sinus of a canine model [38]. These characteristics are summarized in Figure 3.

## 5. Fibrin Handling for Bioactive Compounds Delivery

### 5.1. Tuning Fibrin Intrinsic Characteristics

Bioactive compounds including growth factors, cytokines, drugs, and nucleic acids can be easily entrapped in the fibrin matrix by mixing them with fibrinogen or thrombin. In general, fibrin matrix remains porous regardless the conditions and/or thrombin and fibrinogen ratios. However, increasing fibrinogen and/or thrombin concentrations densifies the structure of a fibrin gel and reduces its degradation rate, thus slowing down the diffusion of loaded molecules (e.g., FGF) [39].

In addition, the release profile of active compounds is affected by their physicochemical properties including partition coefficient, size and functional groups. For example, hydrophilic molecules readily leach out (e.g., erythromycin, mitomycin C, fluorouracil etc.), while hydrophobic molecules, or those which display a strong chemical affinity for the matrix components, are released more gradually (e.g., streptomycin, sulfamethoxazole-trimethoprim, cefazolin, enocitabine etc.) [40,41,42,43]. Thus, some compounds need to be modified chemically to become more hydrophobic, resulting in a prolonged retention within the matrix. Conversely, a proteolytic degradation may be required to speed up the diffusion process.

**Figure 4:**
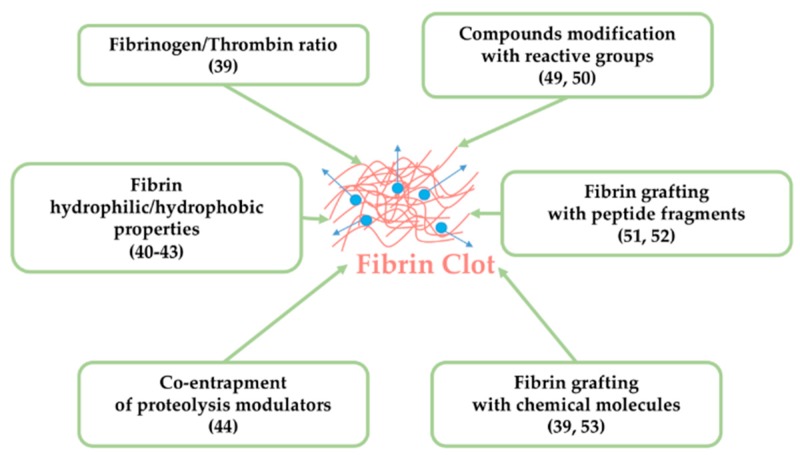
Fibrin and active agents handling for compounds delivery.

Lastly, fibrin matrix proteolysis through the action of thrombin or plasmin induces the release of entrapped compounds. In order to restrict this breakdown of the matrix, the co-entrapment of proteolysis inhibitors (aprotinin, matrix metalloproteinases inhibitors such as galardin) was investigated. Ahmed et al. identified in their model (i.e., chondroprogenitor clonal cell line) the enzymes responsible for fibrin gel breakdown [44]. It appeared that metalloproteinases (MMP-2, MMP-3, MMP-9) were secreted concomitantly with fibrin hydrogels breakdown. They also noted high plasmin activity in the conditioned media during hydrogel breakdown. Then, they demonstrated that aprotinin and galardin, in combination or separately, prevented fibrin hydrogels degradation. Figure 4 summarizes the modifications which can be made on fibrin matrix to regulate the release of entrapped agents.

### 5.2. Modifying Fibrin and/or Active Agents for Efficient Combination and Delivery

In some instances, fibrin exhibits innate affinity for therapeutics and growth factors [45,46,47,48] but in other cases, the covalent attachment of these compounds to fibrin is considered. This generally requires chemical modifications with reactive functional groups (thiols, azides) allowing a stable association [49]. Then, reduction/hydrolysis reactions or enzymatic cleavage mediate the release of active molecules. It was shown that this approach prolongs the delivery phase of growth factors for example [50].

As another illustration, covalently binding of BMP and RGD sequences to fibrin allows interactions with bone marrow stromal cells through integrin surface receptors that triggered downstream signaling pathways [51]. More sophisticated constructions have been tested. Starting from BMP-2, Schmoekel et al. propose a tripartite fusion protein denoted as TG-pl-BMP-2 [52]. Under the control of the blood transglutaminase factor XIIIa, the N-terminal transglutaminase substrate domain (TG) provides a covalent attachment to fibrin during coagulation. In addition, upon the action of cell-activated plasmin, the central plasmin substrate domain (pl) gives a cleavage site for the local release of the attached growth factor from the fibrin matrix. Lastly, the C-terminal human BMP-2 domain displays an osteogenic activity.

As an alternative to active molecules derivatization, fibrin functionalization can also be achieved. For example, to facilitate BMP interaction with a fibrin matrix, Yang et al. grafted heparin onto fibrin, this leading to electrostatic interactions between the negative charges of heparin sulfate groups, and the positive charges of BMPs amino acid residues [53]. Interestingly, heparin presence slowed down fibrin degradation, and consequently the release of loaded BMPs. Similar observations were made with FGF [39].

Despite positive effects reported in literature, there are several drawbacks to fibrin or growth factors/proteins functionalization. First of all, it requires specific skills, and it is time consuming and expensive. Furthermore, proteins functionality can be altered by these modifications. Indeed, covalent binding can mask active sites, thus affecting their bioactivity. As an alternative, non-modified compounds can be internalized into particles, to slow down their release. Wang et al. developed a sophisticated system which could serve to deliver low-molecular weight hydrophilic drugs [54]. They used an antithrombosis drug (i.e., Tirofiban^®^) as a drug model. Drug-loaded liposomes were first encapsulated in a fibrin gel, and then combined to chitosan. To document/characterize the in vitro drug release profile, different parameters were studied including the surface charge of liposomes, the pore size of chitosan structure, and the crosslinking degree of fibrin gels. All these experimental conditions offered different prolonged releases without altering the composite bioactivity. All these modifications are summarized in Figure 4.

## 6. Active Agents Used in Fibrin-Based Delivery Strategies

### 6.1. Active Agents for Bone Infections

Postoperative infections associated with the implantation or the revision of a bone medical device generates undesirable morbidity [55]. In its most extreme occurrences, osteomyelitis, either acute or chronic, can compromise the vital prognosis of the patient. Redl et al. were among the first to investigate fibrin as a carrier for antibiotics [56]. They observed that approximately 85% of the antibiotic was released within 72 h, and that gentamycin for example reduced clot strength by affecting fibrinogen chain-crosslinking. Greco et al. observed similar results in vitro [40]. Tredwell et al. reported then that erythromycin was released slowly over the first 2 hours but then degraded rapidly, whereas cefazolin appeared more stable and was evenly liberated during the first day [41]. Interestingly, release profiles correlated with molecules hydrophobicity and for example, a poorly soluble antibiotic such as tetracycline was retained longer in fibrin network [42].

Regarding in vivo experiments, antibiotics such as vancomycin and sisomicin (a gentamycin analogue), have been associated with fibrin glue and implanted in rats [57,58]. Pharmacokinetic studies revealed a better distribution of drugs in the tissue around the implantation site when compared with intravenous injections. In a model of methicillin-resistant *Staphylococcus aureus* infection, Ozaki et al. reported that fibrin glue provided a sustained release of vancomycin, which was sufficient to prevent the development of severe infections observed in the control group [59]. Interestingly, in the absence of any systemic drug administration, topical administration of antibiotic via their fibrin glue was effective against localized MRSA graft infection. Thus, topical administration of antibiotics may help to treat difficult graft infections and to reduce the use of the systemic route.

Antibiotic resistance is a global health issue which jeopardizes the effectiveness of treatments for a multitude of infections. A recent report published by the World Health Organization points out that the current clinical pipeline is insufficient to tackle the challenge of antimicrobial resistance [60]. Therefore, more innovative long-term strategies are expected, with a view to eliminating pathogens resistance. With this in mind, the use of bacteriophages could be a helpful approach. Bacteriophages can be defined as viruses that specifically infect bacteria. Indeed, bacteriophages are good candidates for antibacterial therapy as they are (i) non-toxic, (ii) highly specific, (iii) able to replicate themselves as long as host bacteria are present, and finally (iv) some of them are able to penetrate and disrupt biofilms, usually characterized by their high resistance to host immune defenses and to antibiotics penetration [61,62]. Considering this powerful tool, Rubalskii et al. investigated the potential of fibrin glue to incorporate and release bacteriophage in an effective manner [63]. Their data showed that the high titers of bacteriophages released for 11 days were effective in killing *Pseudomonas aeruginosa*.

### 6.2. Active Agents for Pain Relief and Cancer Treatment

Postoperative pain after bone reconstruction is another serious complication that can jeopardize surgery global success [64]. As a matter of fact, pain significantly reduces patients’ mobility, delays their functional recovery, extends their hospital stay, and decreases their quality of life and autonomy. In this context, different approaches have been explored to address these issues [65,66,67] and for example, fibrin glue loaded with local anesthetics (lidocaine) was shown to reduce patients’ pain intensity after surgery [67].

In relation with pain but also with structural integrity issues, bone tissue is one of the most favored sites for metastasis of solid tumors (especially for prostate, breast, lung and kidney cancers), and these lesions are preferentially localized in spine and pelvic bone [68]. For example, up to 70% of all patients diagnosed with a breast cancer will develop bone metastases. These lesions ultimately cause dramatic tissue defects that compromise bone structural integrity. This results in severe bone pain and instability, fractures, spinal cord compressions, hypercalcemia and bone marrow aplasia. Thus, among therapeutic approaches, the local release of chemotherapeutic drugs from fibrin gel was investigated. The comparative analysis of various chemotherapeutic drugs release profiles revealed a short release period [43]. Thus, a burst release of doxorubicin from a fibrin gel was observed while sodium alginate addition tended to extend release duration over several days [69]. In the same way as antibiotics, chemotherapeutic drugs release rate correlates with molecules hydrophobicity, suggesting that the release of lipophilic drugs may require matrix degradation, whereas water-soluble agents readily diffuse out.

### 6.3. Growth Factors and Cytokines

As largely described, growth factors are strongly involved in the process of bone regeneration as recapitulated in Table 1 [70,71,72,73,74,75,76,77,78,79,80,81,82,83]. They initiate the healing process by providing at the injury site signals that are essential for the recruitment of inflammatory and progenitors cells. Among these growth factors, bone matrix proteins (BMPs), including BMP-2 and 7, received special attention due to their potent osteoinductive properties. In fact, they stimulate (i) the migration of mesenchymal stem cells to bone-forming sites, (ii) their differentiation into an osteoblastic lineage, and (iii) bone tissue regeneration. However, the adjustment of BMPs local concentration for a sufficient period of time remains a challenge. Indeed, a short burst of BMPs not only causes deleterious effects on tissue but also requires a more frequent administration, which is costly. To address these problems, the fibrin matrix was tested to deliver BMPs and for example, BMP-2 was mixed with fibrinogen and thrombin [74] and then injected into a rat femoral defect. When compared with a collagen sponge, and despite a seven-fold lower content in growth factor, the BMP-2-loaded fibrin matrix displayed a superior osteogenic capacity. Interestingly, in a rat calvarial defect model, the combination of BMP-2-loaded fibrin with calcium phosphate particles improved fibrin composite action on bone repair [75]. In an effort to limit BMPs diffusion toward undesired sites, Patel et al. encapsulated BMP-2-loaded collagen sponges in fibrin glue. Unfortunately, BMP-2 still could diffuse through fibrin pores, probably because of its small size and its low affinity for fibrin [76]. By contrast, in a rabbit spinal fusion model, conjugating heparin to fibrin delayed the release of BMP-2, and resulted in biological results similar to those obtained with autograft, which is the gold standard for bone reconstruction [77]. Lastly, it was shown that the co-delivery of BMP-2 and platelet-derived growth factor (PDGF) enhanced vascularization and bone formation in mouse calvarial defects [78].

Vascularization plays a critical role in bone fractures healing. Vessels not only provide oxygen and a nutritional support for newly formed tissues, but they also constitute a network allowing the circulation of inflammatory cells, progenitors and growth factors such as VEGF. VEGF is considered as the most important angiogenic factor involved in bone formation [70]. By stimulating the proliferation and migration of endothelial cells, VEGF promotes blood vessels formation, and thus the recruitment and the survival of bone forming cells. Burkhardt et al. have reported the physiological importance of blood clots to create a pro-angiogenic environment which favored healing response [79].

Numerous delivery systems, for VEGF alone or combined with other growth factors, have been developed. For example, in a canine model of femoral neck fracture, bone delivery of VEGF incorporated into fibrin-suspended PLGA microspheres induced significant osteogenic and angiogenic effects [80]. Regarding the release kinetic profiles, a sequential release of two growth factors was achieved using a VEGF-loaded gelatin hydrogel surrounding a poly(propylene) core, which included BMP-2-loaded PLGA (polylactic-*co*-glycolic acid) microspheres [81]. Indeed, in a rat model, an initial burst release of VEGF was observed during the first three days, whereas BMP-2 displayed a sustained release over the full 56-day implantation period. Similarly, using a dual delivery system of BMP-2 and VEGF, Subbiah et al. reported a beneficial effect on both angiogenesis and osteogenesis [82].

### 6.4. Genes

Severe postoperative complications, including osteolysis, overgrowth and pseudarthrosis have been observed following the administration of highly concentrated BMP-2 protein [84]. With this respect, the use of coding DNAs under the control of suitable promoters, transfected or transduced in relevant cells, appeared as an alternative way to supply growth factors. For that purpose, incorporating viral and non-viral vectors into fibrin glue was investigated to deliver genes.

Considering that transcriptional activity declines rapidly, a sustained release is required to achieve long-term effects. Ionic interaction of DNA with the matrix may favor retention of the vector [85]. A preliminary study in a rabbit ear ulcer model investigated the ability of fibrin to deliver a low single dose of viral vector (i.e., adenovirus encoding beta-galactosidase) to a wound site, without compromising transfection efficiency [86]. Although transgene expression was enhanced in the fibrin containing adenovirus group at seven days, no difference was measured at 14 days when compared to the control group, and the presence of the virus did not cause any unfavorable inflammatory response. Des Rieux et al. reported that non-viral vectors could be efficiently entrapped within a fibrin hydrogel, and that after one day in solution, more than 70% of the entrapped DNA was still retained within the gel, with a sustained release observed for at least 19 days [87]. Recently, Bara et al. combined a doxycycline-inducible adenoviral vector with mesenchymal stem cells, fibrin and calcium phosphate granules [88]. Although only low levels of BMP-2 protein secretion were measured in vitro (ng/mL), in vivo implantation into a rat bone critical defect for 12 weeks revealed an improvement of bone regeneration. By suggesting that low levels of growth factors could be effective for promoting an appropriate tissue response, this result may validate the delivery gene method as a relevant alternative approach to the use of peptides or proteins.

### 6.5. miRNAs/siRNA

Small interfering RNA (siRNA) is defined as a gene-silencing mechanism by which post-transcriptional gene silencing can occur. Delivering a siRNA alone is very complicated because of negative charges preventing its internalization through the cell membrane. Moreover, siRNA molecules are susceptible to degradation. To circumvent these limits, various approaches including viral and non-viral vector-mediated delivery have been explored [89]. For example, the noggin siRNA, an antagonist to BMP-2, was packaged into a non-viral vector and then embedded into a fibrin gel [90]. In vitro studies showed, after a 48 h treatment, a significant intracellular uptake of siRNA in over 98% of osteoblasts. These low doses of noggin siRNA were sufficient to significantly reduce noggin mRNA levels in exposed cells, without altering their viability.

## 7. Conclusions and Perspectives

Despite the benefits that minimally invasive osteosynthesis and surgery have brought to bone repair, there are still many circumstances where achieving bone healing remains challenging. In this context of bone reconstructive surgery, fibrin has shown a high potential. This was not only as a source of growth factors with osteoconductive properties, but also as a delivery system for active biological compounds that could further improve bone tissue regeneration or more generally prevent/treat skeletal disorders. However, clinical effectiveness is still controversial, depending on the variability of fibrin preparation protocols used, and the limited number of patients included in the trial [91,92]. For example, no positive effects of fibrin have been notified in patients after total knee replacement surgery [93], whereas fibrin provided beneficial effects in improving alveolar preservation on extraction sockets and around dental implants [94]. Hence, further well-designed clinical trials are expected to conclude on the effectiveness of fibrin.

Nowadays, innovative approaches are explored to optimize fibrin properties in terms of mechanical and biological responses. Most of studies are focused on improving its mechanical strength through the addition of other polymers or biomaterials, including calcium phosphates (CaP). Interestingly, CaP-based biomaterials not only provide sufficient mechanical properties, but also a favorable support for cell attraction, proliferation and activity [2,3,95]. Indeed, as compared to fibrin alone, cell proliferation strongly increased in the presence of CaP-based materials/fibrin composites [96].

In addition, cells are necessary to support bone tissue healing/regeneration, and thus might confer to fibrin supplemental osteogenic properties. In this attempt, fibrin can serve as delivery system for different cell types including fibroblasts [97], osteoblasts [98], dental stem cells [99] and mesenchymal stem cells isolated from various sources [23,100]. Recently, embryonic and induced pluripotent stem cell-derived progenitor cells have been uniformly mixed within fibrin [101]. Interestingly, high cell seeding densities have been reached successfully, this resulting in good viability, proliferation and differentiation properties [22,25]. However, further experiments are required to study the impact of fibrin intrinsic properties (i.e., composition/structure/stiffness etc.) on cell behavior. Understanding and controlling these interactions might encourage the use of fibrin as a carrier rather than systemic injections for cells administration.

At the present time, a growing interest focuses on fibrin-based nanostructures to provide a suitable natural matrix environment supporting bone regeneration. These nanoscaffolds are explored in tissue regenerative medicine to deliver drugs, active biomolecules, cells and genes. For example, Praveen et al. prepared fibrin nanoconstructs (FNCs) including nanotubes and nanoparticles, through a modified water-in-oil emulsification-diffusion route, and without the use of any surfactants [102]. Surprisingly, these constructs displayed a high resistance to aggregation, and excellent temperature stability up to 200 °C. Tacrolimus, an immunosuppressive drug, was encapsulated into these fibrin nanostructures. A sustained and complete drug release has been observed over a period of one week. Further in vivo experiments are expected to prove the therapeutic efficiency of these constructs. Nanofibrin has been also incorporated into a chitin-CaSO4 injectable gel to improve its rheological behavior as well as its pro-angiogenic properties [103]. Indeed, rheological studies reported that the composite gel including nanofibrin was a shear thinning gel, with an elastic modulus increased by 1.67-fold over the chitin control. In vitro studies performed on rabbit adipose-derived mesenchymal stem cells (rASCs) demonstrated that nanofibrin addition tends to enhance rASCs activities. In another study, the authors addressed the issue of vasculature stimulation through the design of a nanocomposite injectable hydrogels [104]. These gels were based on chitin and poly (butylene succinate) (PBSu) loaded with fibrin nanoparticles (FNPs) and magnesium-doped bioglass (MBG). FNPs were expected to promote vascularization, whereas MBG might stimulate early osteogenesis. Authors demonstrated that composites containing 2% FNPs and 5% MBG displayed good rheological properties, injectability, temperature stability, biomineralization and protein adsorption without inducing cytotoxicity. They also reported an enhancement of both pro-angiogenic and osteoinductive properties.

Considering bone repair, smart solutions including nanocomposites are emerging. However, keeping in mind a potential strong impact on cell responses, and thus on bone tissue full rehabilitation, these last generation biomaterials require further in-depth analysis.

## Figures and Tables

**Table 1:** Main functions of growth factors used in bone tissue engineering [70,71,72,73,74,75,76,77,78,79,80,81,82,83].

Growth Factor	Functions for Bone Regeneration
BMP (Bone matrix protein)	Recruiting MSCs to the injury site Triggering osteogenic differentiation of osteoprogenitors
FGF (Fibroblast growth factor)	Stimulating proliferation of endothelial cells and osteoblasts Promoting angiogenesis from preexisting vasculature Regulating MSC, endothelial cells, fibroblasts and osteoblasts
PDGF (Platelet-derived growth factor)	Promoting chemotaxis of MSC, macrophages, neutrophils, fibroblasts and osteoblasts Modulating collagenase synthesis and collagen secretion
VEGF (Vascular endothelial growth factor)	Stimulating proliferation and migration of endothelial cells to regulate angiogenesis during bone formation Promoting recruitment and survival of bone forming cells
TGF-β (Transforming growth factor β)	Stimulating proliferation of MSC, endothelial cells, fibroblasts and osteoblasts Inhibiting macrophage and lymphocyte proliferation Regulating collagenase synthesis and collagen secretion
